# Integrated analysis of Hashtgerd plain deformation, using Sentinel-1 SAR, geological and hydrological data

**DOI:** 10.1038/s41598-022-25659-4

**Published:** 2022-12-13

**Authors:** Mahdi Khoshlahjeh Azar, Siavash Shami, Faramarz Nilfouroushan, Maryam Salimi, Mahdieh Ghayoor Bolorfroshan, Mir Amir Mohammad Reshadi

**Affiliations:** 1 Department of Remote Sensing and Photogrammetry, Faculty of Geodesy and Geomatics Engineering, K. N. Toosi University of Technology, Tehran, Iran; 2 Department of Geodesy, Faculty of Geodesy and Geomatics Engineering, K. N. Toosi University of Technology, Tehran, Iran; 3 Department of Computer and Geospatial Sciences, University of Gävle, Gävle, Sweden; 4 Geodata Division, Department of Geodetic Infrastructure, Lantmäteriet, Gävle, Sweden; 5 Department of Minerals and Groundwater Resources, Faculty of Earth Sciences, Shahid Beheshti University, Tehran, Iran; 6 Department of Geography, Ferdowsi University of Mashhad, Mashhad, Iran; 7 Ecohydrology Research Group, Department of Earth and Environmental Sciences, University of Waterloo, Waterloo, ON Canada

**Keywords:** Environmental sciences, Natural hazards

## Abstract

Due to its proximity to Tehran, the Hashtgerd catchment in Iran is an important region that has experienced alarming subsidence rates in recent years. This study estimated the ground surface deformation in the Hashtgerd plain between 2015 and 2020 using Sentinel-1 SAR data and InSAR technique. The average LOS displacement of the ascending and descending tracks was − 23 cm/year and − 22 cm/year, respectively. The central area of the plain experienced the greatest vertical subsidence, with a more than − 100 cm cumulative displacement. The Karaj-Qazvin railway and highway that pass through this area have been damaged by subsidence, according to an analysis of profiles drawn along the transportation lines. The southern sections of Hashtgerd city have experienced a total displacement of − 30 cm/year over the course of about 6 years. The relationship between changes in groundwater level and subsidence rate in this region was examined using piezometer and precipitation data. Geoelectric sections and piezometric well logs were also utilized to investigate the geological characteristics of the Hashtgerd aquifer. According to the findings, the leading causes of subsidence were uncontrolled groundwater abstraction. This research highlights the need to comprehend the spatial distribution of confined aquifers and their effect on subsidence, which can aid in the development of a suitable management strategy to restore these aquifers.

## Introduction

Land displacement is caused by a combination of anthropogenic and natural factors, which can result in loss of human life and economical resources. The subsidence of California in the United States^1^, Beijing in China^2^, Maceió in Brazil^3^, Karachi in Pakistan^4^, Uppsala and Gävle in Sweden^5,6^, and Mexico City in Mexico^7^ are all well-known examples of such events triggered by groundwater abstraction, geological causes, and other human factors^8–10^. Many Iranian plains, including Hamadan^11^, Rafsanjan^12^, Kashan^13^, and Qazvin^14^ plains, have experienced subsidence in last decades due to the over-extraction of groundwater^15^. The Hashtgerd plain, located 70 km west of Tehran, has experienced vast amount of subsidence which has resulted in threats such as road distortion, building settlement, power tower displacement, and ground surface fissures.

Despite their high accuracy, traditional displacement monitoring methods, such as leveling and GNSS are too time-intensive, laborious, and costly. The Interferometric Synthetic Aperture Radar (InSAR) method, on the other hand, with its high coverage and millimeter-level accuracy, has evolved into a successful tool for detecting ground deformation^16,17^. However, topographic, orbital, and atmospheric errors, as well as other noises, limit the accuracy of this technique. Multi-temporal InSAR techniques such as PS^18–21^ and SBAS^22–24^ can get over these restrictions. The PSInSAR is commonly appropriate for urban areas with strong density of persistent scatterers (PSs). As a result, the SBAS approach can be an effective tool in areas dominated by agricultural lands, where PS densities are low. The SBAS technique, which is founded on a small temporal and perpendicular baseline, maximizes coherence by reducing unwrapping and uncorrelated noise errors^23^. As a result, the NSBAS algorithm has been proposed in recent years to address this limitation^8,25^.

In a study conducted by Mehrnoor et al., (2022) to investigate the risk of subsidence in the Hashtgerd plain, support vector machine (SVM) and weighted overlay index (WOI) models were used to classify subsidence risk zones based on 19 geological, hydrological, hydrogeological, and environmental criteria weighted using the best-to-worst method (BWM). The results of BWM indicate that groundwater abstraction, lithology, and groundwater depletion are the most influential factors in this area's subsidence^26^. Taesiri et al., (2020) created a map by combining indices of related active tectonics (IRAT) and weighted, combined morphometric indices, demonstrating a high level of tectonic activity at the center of the Hashtgerd plain. Near the city of Hashtgerd, a tectonic survey identified a crushed zone caused by collisions between active tectonic plates. The results clearly demonstrate intense activities at the center of the plain, with a significant correlation between the crushed zone and the hypocenter of large earthquakes^27^.

In Iran's Alborz province, groundwater level decrease and the resulting gradual subsidence of more than 20 cm per year have become one of the greatest environmental concerns. On the other hand, potential future catastrophes will consequence from the province's excessive extraction of groundwater resources. As the most important agricultural region in the province of Alborz, agricultural practices in the Hashtgerd plain consume 87.23% and 90% of the region's surface and groundwater resources, respectively Shabani Rouchi et al., (2022)^28^. In this regard, Hanifehlou et al., (2022) examined the groundwater supply and demand balance in the Hashtgerd plain, one of the most important agricultural centers in Iran. In this study, which used the WEAP model to propose managerial scenarios applicable to the situation at hand, 2020 was chosen as the base year for the prediction of the pattern through 2050, taking climate change and land use type into account. In the most severe cases, when climate change and land use type are considered together, the average groundwater level decline will reach 58 m per year, depleting more than 50 percent of the aquifers in the region^29^.

InSAR based research has been carried out in recent years to analyze ground displacement in the Hashtgerd plain. For example, Dehghani et al., (2008) investigated ground surface displacement in this plain using Envisat satellite images and the DInSAR technique. The findings revealed a 7 cm subsidence in 35 days^30^. Ashrafianfar et al. (2009) studied land surface deformation in the plain using 10 interferograms generated from Envisat satellite imagery from 2003 to 2004 and 11 interferograms from 2007 to 2008. The results showed a subsidence of 8–14 cm in 2004 and 20 to 24 cm in 2008^31^. In a similar study, Haghighatmehr et al. (2012) monitored ground surface deformation in Hashtgerd plain in 2012 using Envisat satellite imagery and the SBAS algorithm. The results revealed monthly subsidence of 47 mm over a four-month period in 2008, introducing groundwater over-abstraction as the most significant contributor to subsidence^32^. Ashrafianfar et al. (2014) investigated the subsidence level in Hashtgerd plain using PSInSAR and ALOS images in conjunction with the Envisat ASAR data and discovered a subsidence rate of about 14 cm from 2003 to 2008^33^.

The primary goal of this study is to broaden the scope of the previous studies by using the NSBAS InSAR technique on Sentinel-1 data in both ascending and descending tracks to generate two-dimensional displacement maps of the Hashtgerd aquifer and improve the characterization of spatial and temporal displacement behaviors. Using the NSBAS method in the Hashtgerd plain, which has an extensive agricultural land cover, ensures reliable results regarding the lack of good coherence in agricultural land cover. On the other hand, the presence of multiple masking criteria and the elimination of noisy pixels ensures the high reliability of the remaining pixels. These benefits, which have been undervalued in previous works, are useful when studying such a plain. Using the SAR data collected between 2015 and 2020, the NSBAS algorithm is applied to 117 and 108 Sentinel-1 images for ascending and descending tracks, respectively. Furthermore, the relationship between land displacement and groundwater changes was investigated by modeling the water level data obtained from piezometers. The rate of aquifer recharge was monitored using precipitation data from the CHIRPS satellite. Data from exploratory wells and geoelectric sections were used to identify the sediment type and thickness as well as the effect of different aquifers of the region on subsidence in the Hashtgerd catchment.

## Study area and geological setting

**Figure 1:**
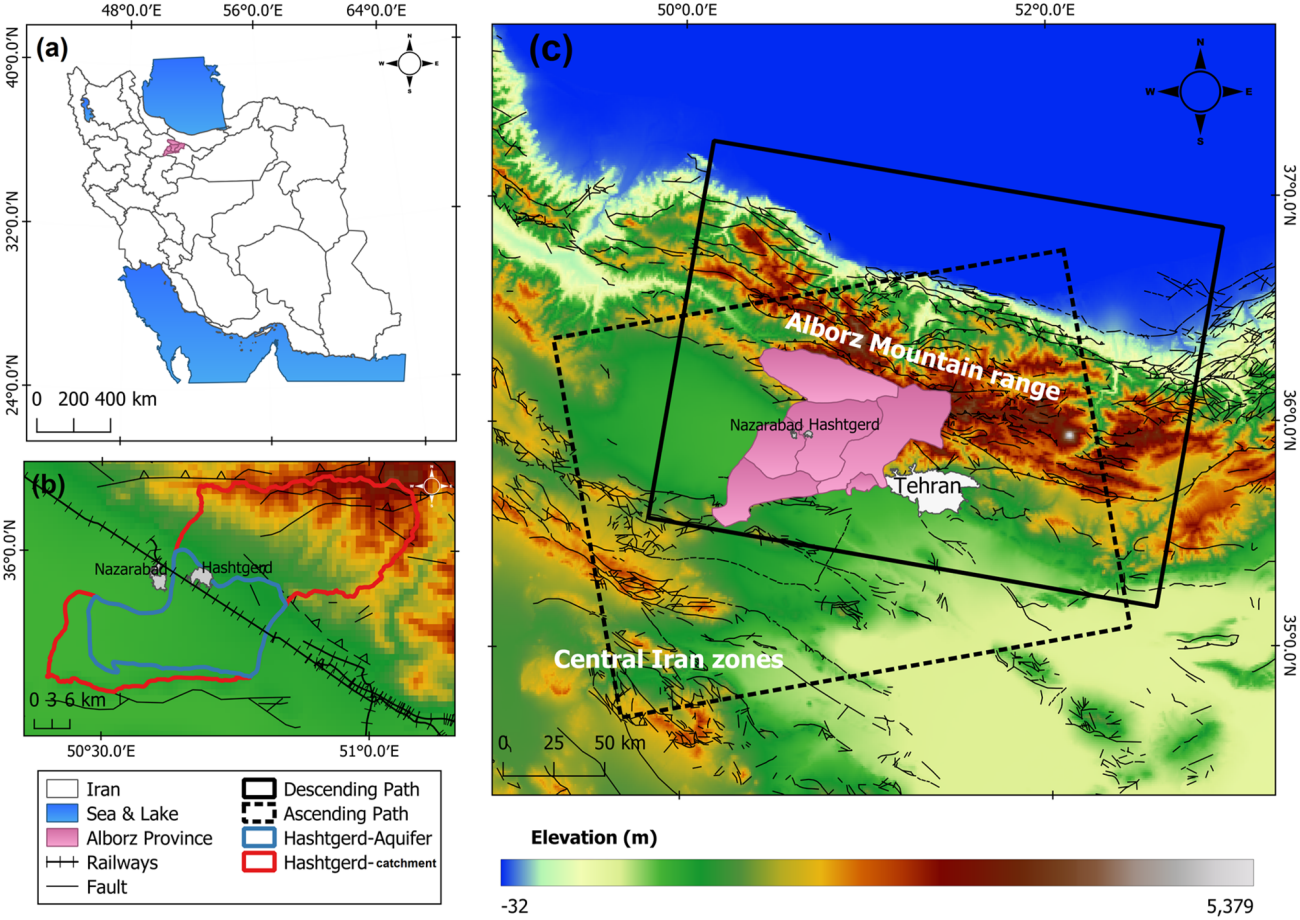
Study area, (**a**) location of Alborz province in Iran, (**b**) position of the Sentinel-1 images' ascending and descending tracks on digital elevation model (DEM) of the region, (**c**) Hashtgerd catchment and aquifer and faults of the region under study. This figure was created using the QGIS version Desktop 3.18.1 software (https://qgis.org/en/site/).

**Figure 2:**
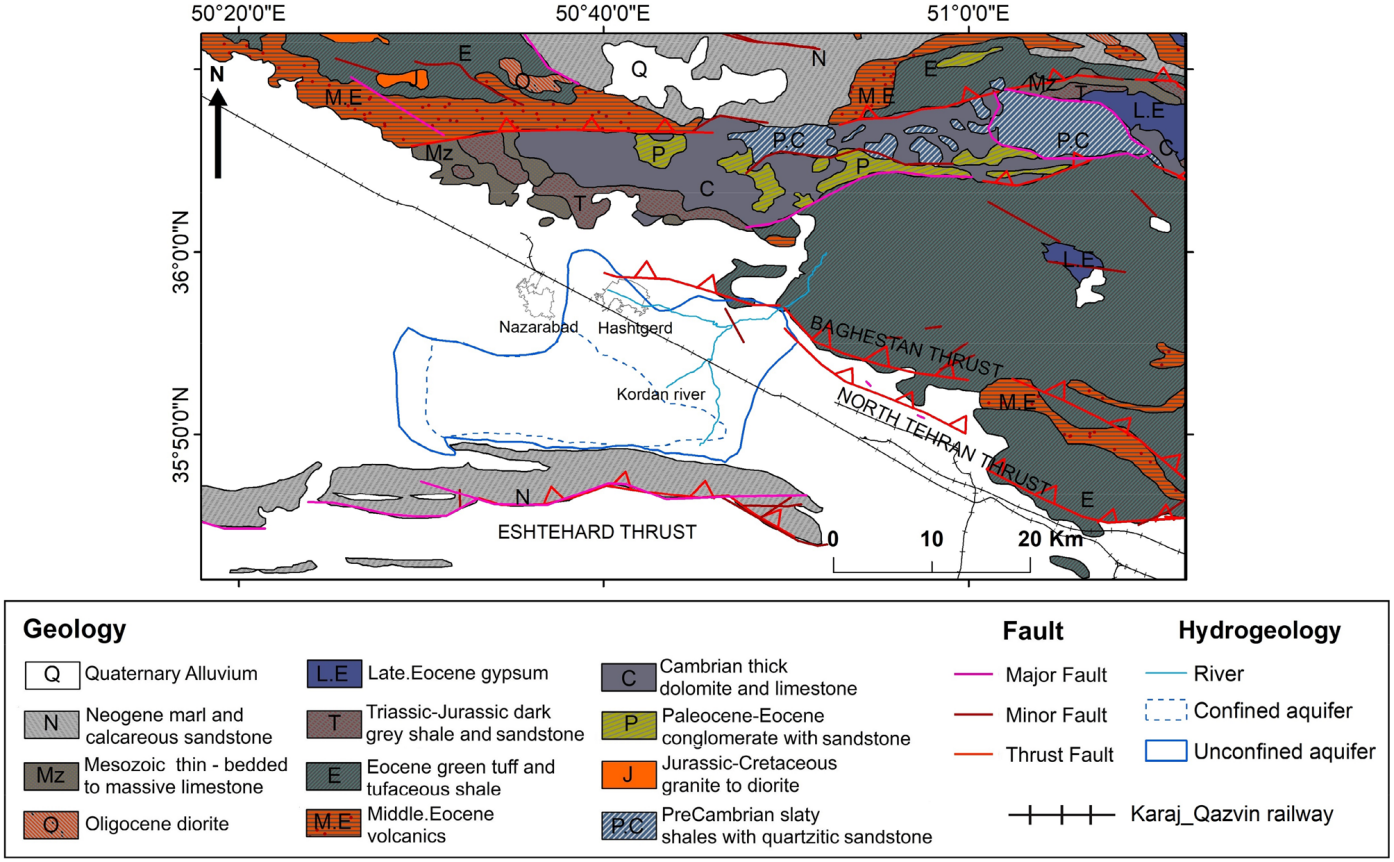
Geological map of the research region (Geological Survey of Iran). This figure was created using the QGIS version Desktop 3.18.1 software (https://qgis.org/en/site/).

The Hashtgerd catchment, with an area of 1276 square kilometers, is located in Alborz province, west of the capital city, Tehran (Fig. 1). The population is increasing in this area because of the fertile soil, semi-wet climate, constant flow of the Kordan River and proximity to Tehran^33^. The Hashtgerd catchment is situated on two alluvial fan and plain units in Iran's central Alborz geological zone and the northern margin of the Dasht-e Kavir. According to the geological map, alluvial fans are spread across Eocene volcanic rocks, Paleozoic shales and sandstones, and Mesozoic limestones (Fig. 2). Also, Hashtgerd plain, with an area of 752 km^2^, is located on Quaternary alluvial sediments. There are aeolian sands and Saline fields on the southern margin of the Hashtegerd plain^34–36^.

The geophysical and geological studies indicated that the thickness of the alluvium in this plain ranges from zero to about 300 m, with the minimum thickness on the bedrock of the Pliocene conglomerates in the northern heights and the maximum in the center of the plain^37^.

The region's thrust and reverse faults run east–west parallel to the Alborz Mountain range, and the Hashtgerd plain has been compressed due to its location between the Alborz Mountain range and central Iran zones, and it has been depressed by the Northern Tehran thrust fault and the Eshtehard thrust fault in the south^35^. Hydrologically, this region is located in the northern area of the Iran Salt Lake catchment^34^. Kordan River enters Hashtgerd plain from northern heights and is the primary source of aquifer recharge.

In the past, the water erosion of Kordan River caused deposited coarse grain sediments in the plain's northern, fine sediments in the central, and silty-clay sediments in the southern areas of the plain.

An unconfined aquifer is formed in the coarse sediment areas of alluvial fans, and semi-confined and confined aquifers are formed in fine-grained sediments of the plain^35^. The Hashtgerd plain aquifer, covering 410 km^2^, is located in the central and western areas of the region. There are 5034 water wells in the area, the majority of which are exploited for agricultural practices in the plain's center.

### Datasets

**Figure 3:**
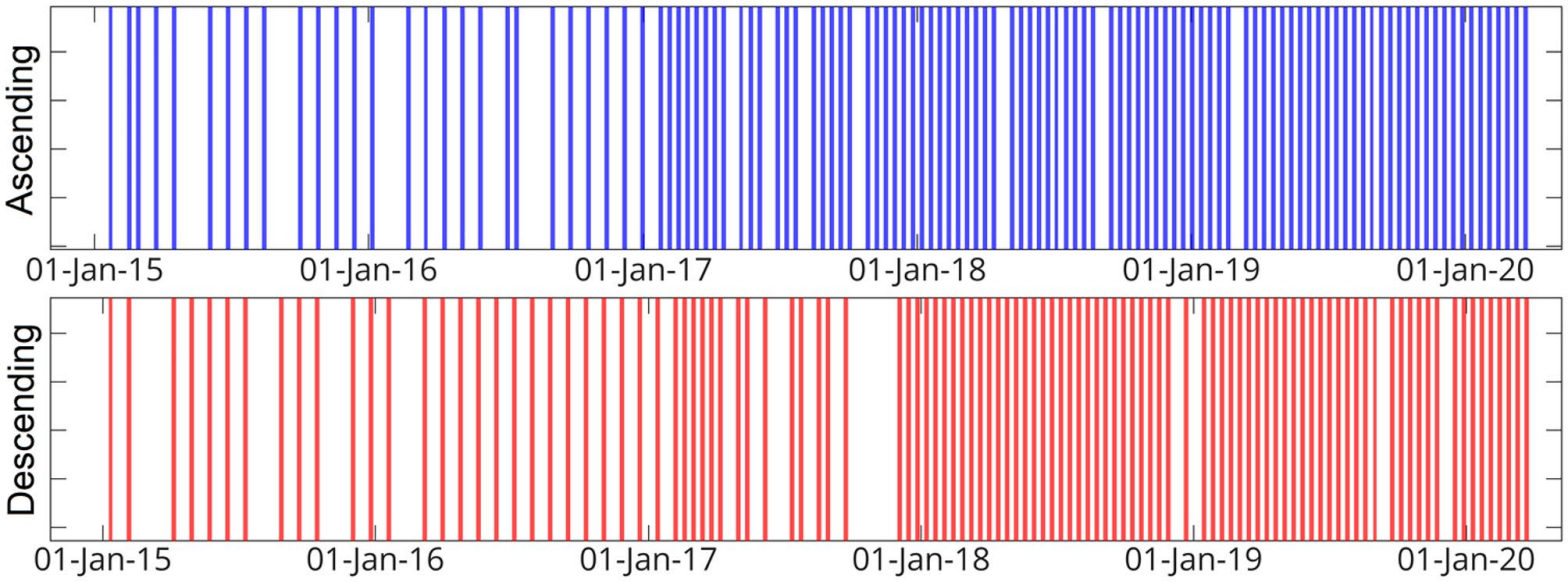
Temporal sampling of sentinel-1 images for InSAR analysis in the (**a**) ascending (blue), and (**b**) descending track (red).

There were 117 ascending track images of the Sentinel-1 satellite from the timespan between 23 January 2015 and 21 April 2020, and 108 descending track images between January 12, 2015 and April 22, 2020. Figure 3 depicts the number and density of images used in the research.

Hashtgerd station (see Fig. 6) is one of the permanent GNSS stations established by National Cartographic Center of Iran for mapping and geodynamic applications. Since 2006, the station has collected the data and its coordinates time series were used here to validate our InSAR results. Piezometer data from 2014 to 2018 were also used to quantitatively evaluate the relationship between displacement yielded by the InSAR method and groundwater-level fluctuations. To explore the spatial correlation of deformation velocity map derived from InSAR analysis, the data was used to map the amount of decline in groundwater level. Sentinel-1,2 satellite images were also used to extract the land use map in Hashtgerd plain and explore the correlation between subsidence and land use. To investigate subsurface events in the Hashtgerd catchment, two types of data were used, namely, exploratory well data and geoelectric profiles. Geophysical studies were conducted using the geoelectric method, which included 46 electrical soundings on 6 profiles, four of which were in the study area. The type and thickness of sediments, bedrock material, and different aquifers were determined using log data from five exploratory wells.

### Methodology and InSAR processing

**Figure 4:**
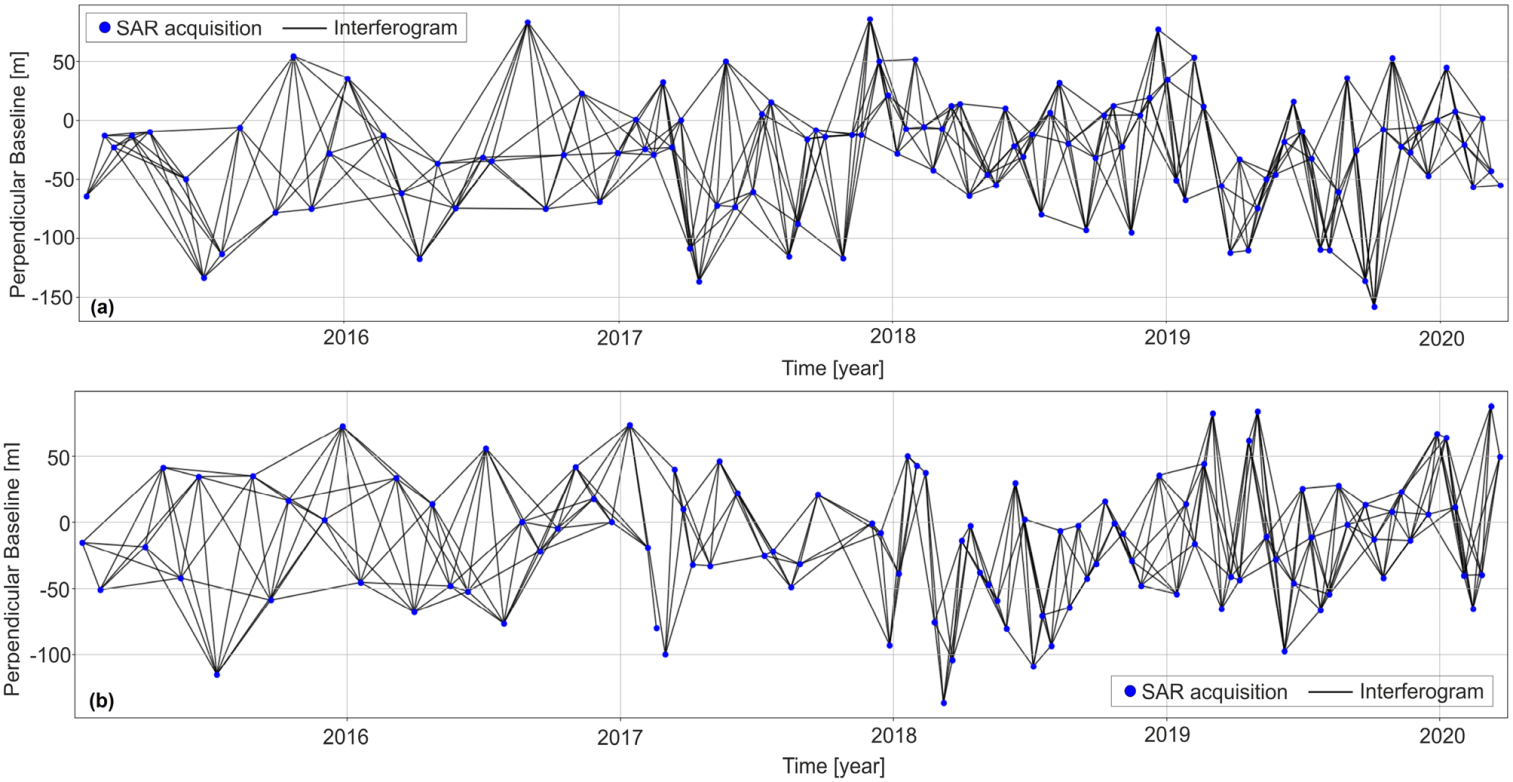
The perpendicular and temporal baseline network for the (**a**) ascending and (**b**) descending tracks.

Unwrapped interferograms and coherence generated by Sentinel-1 images^38^, which are produced using Gamma software using a multiple-looking factor of 20 and 4 for range and azimuth, respectively^39,40^, were used as primary data for our analysis. The wrapped interferograms were unwrapped using SNAPHU software^41^. The LiCSBAS software was used in this study to process the time series of radar images^42–44^. This study employed 448 interferograms for ascending tracks and 370 interferograms for descending tracks. A threshold of 0.3 was used to assess the quality of the interferograms based on the mean value of coherence. With a value lower than this threshold, no ascending or descending track interferograms were detected to be removed from the network. Unwrapping errors could may possibly persist in unwrapped interferograms, resulting in a remarkable bias in estimating deformation rate so the interferograms that still contained unwrapping error were identified and excluded from further processing using root mean square (RMS) for the loop interferograms with a 1.5 (rad) threshold. Then a stable reference point is chosen at Lat: 35.993 and Lon: 50.841. As a result, 10 and 11 interferograms from the ascending and descending tracks, respectively, were opted out. The endmost interferograms network for the ascending and descending tracks was established by removing the undesirable interferometers, as demonstrated in Fig. 4.

A Small Baseline inversion (SB) was conducted on the interferograms graph to evaluate the deformation value changes of the pixels over time in the satellite's line of sight (LOS). At this point, the initial interferograms were inversed using the NSBAS algorithm to secure time series displacement. The NSBAS algorithm was chosen because of the large amount of agricultural land-cover in this area. The standard deviation (STD) of displacement velocity was calculated using the Bootstrap method^45^. Despite the removal of undesirable interferograms in the previous steps, the pixels in some interferograms may still contain noise. As a result, nine indicators proposed in Morishita et al., 2020 were used for masking noisy pixels ^42^, with pixels associated with values less than the threshold being masked. The orbital error, as well as tropospheric and ionospheric noise, were removed from the time series in the final stage using a spatial–temporal filter^20,46^. After obtaining displacement velocity maps for both tracks in the direction of the LOS, and regardless of the displacement in the north–south (NS) direction, the displacement velocity in the perpendicular and east–west (EW) directions can be determined using Eq. (1)^12,47^:

1 $$\left(\begin{array}{c}{V}_{LOS}^{a}\\ {V}_{LOS}^{d}\end{array}\right)=\left(\begin{array}{cc}\mathit{cos}{\theta }^{a}& -{\mathrm{cos}\alpha }^{a}\mathit{sin}{\theta }^{a}\\ \mathit{cos}{\theta }^{d}& -{\mathrm{sin }a}^{d}\mathit{sin}{\theta }^{d}\end{array}\right)\left(\begin{array}{c}{V}_{V}\\ {V}_{EW}\end{array}\right)$$

where $\theta$ is the incidence angle, α is the azimuth angle, $d$ and $a$ denote the descending and ascending tracks, respectively.

**Figure 5:**
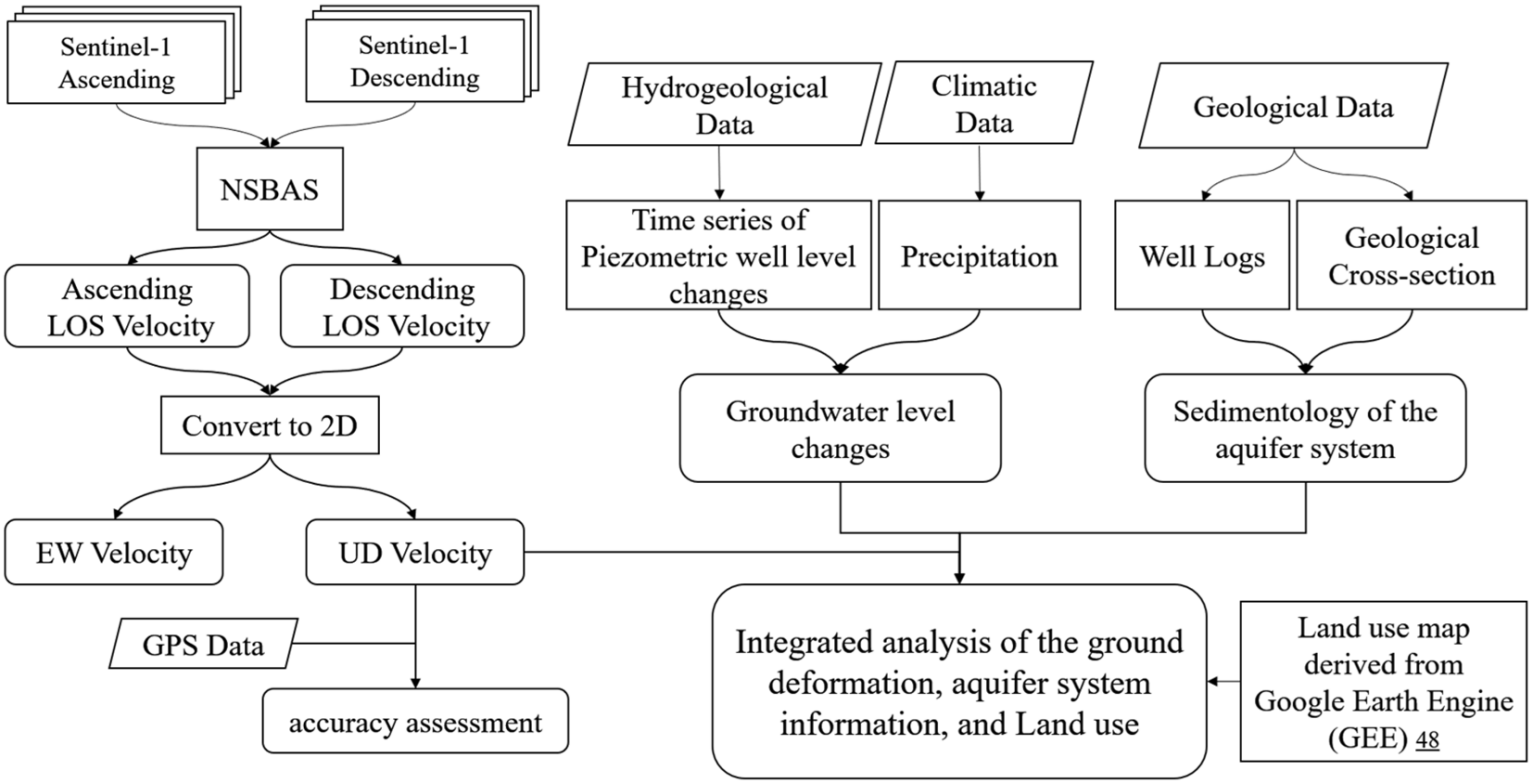
Processing and analysis steps for the data used in this study.

The results of the displacement velocity in the up-down (Vertical) and EW directions in the Hashtgerd plain were then validated using data from the GNSS station, and the effect of changes in the region's hydrogeological status and geological structures on subsidence was investigated. Figure 5 depicts the flowchart of the processing procedure implemented in this study. To determine the relationship between subsidence location and land cover, Iran's land cover map produced by Ref.^48^ in which Google Earth Engine was utilized. This map was processed in 13 classes with a spatial resolution of 10 m using the object-based random forest (RF) algorithm on Sentinel-1 and 2 satellites data, achieving an overall accuracy (OA) of 95.6% and a Kappa Coefficient (KC) of 0.95, which is adequate for comparing the spatial relationship between displacement and land cover.

### InSAR results

**Figure 6:**
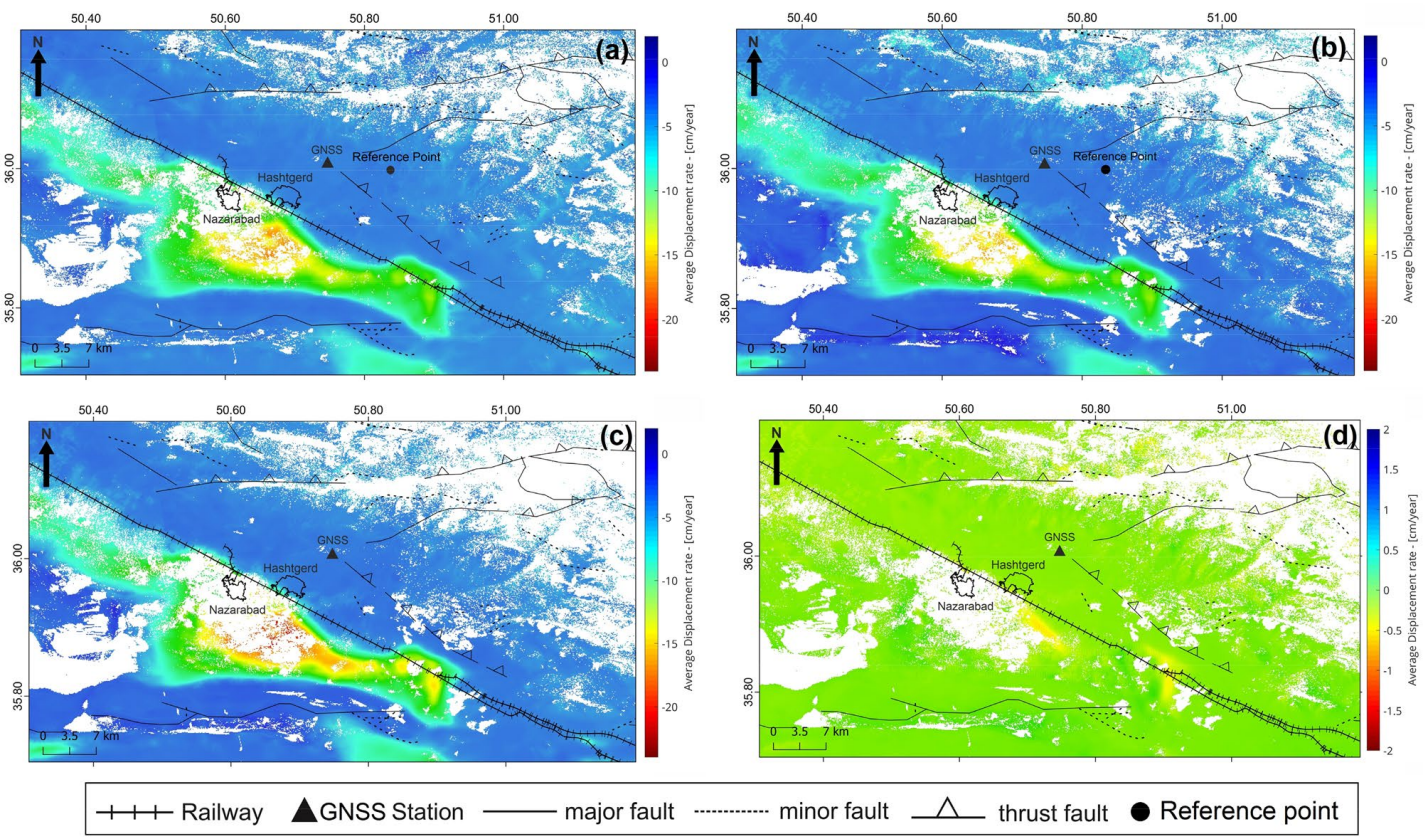
Map of ground surface displacement velocity in (**a**) ascending track (**b**) descending track west direction-displacement velocity in (**c**) vertical direction and (**d**) east. This figure was created using the QGIS version Desktop 3.18.1 software (https://qgis.org/en/site/).

Figure 6 depicts the average displacement velocity obtained for the Hashtgerd plain from 2015 to 2020 using the NSBAS algorithm. The central areas of this plain have the greatest displacement in the LOS direction. The maximum subsidence rate for the ascending and descending tracks are − 23 and − 22 cm/year, respectively. The vertical and horizontal displacement maps were produced using Eq. (1) while combining the results of ascending and descending tracks yielded the 2D displacement rate. According to the findings, the maximum vertical subsidence in this plain is − 30 cm/year. Furthermore, in most areas of the plain, the east–west displacement rate was less than ± 1 cm/year. The maximum standard deviation obtained from processing of ascending and descending tracks is 2.7 mm/year.

## Discussion

### Land subsidence and groundwater level fluctuations

**Figure 7:**
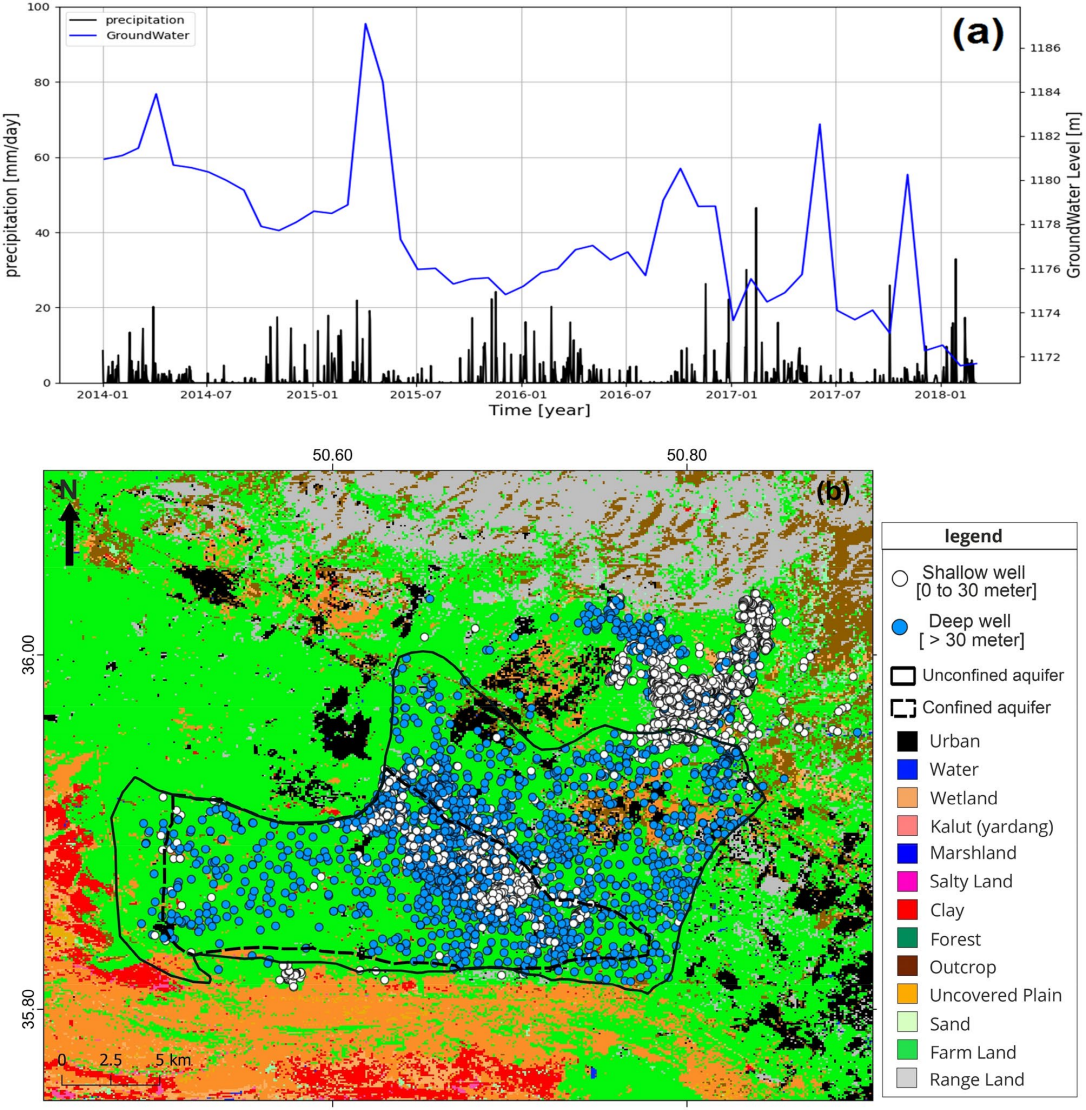
(**a**) Hydrograph derived from average total water level in piezometers and average daily rainfall using CHIRPS dataset in GEE between 2014 and 2018 for Hashtgerd catchment, and (**b**) land use map yielded by GEE and location and distribution of deep and shallow wells. This figure was created using the QGIS version Desktop 3.18.1 software (https://qgis.org/en/site/).

The hydrograph obtained from groundwater level fluctuations in 23 piezometers shows that the groundwater level in Hashtgerd plain dropped more than 9 m between 2014 and 2018, from 1181 m in 2014 to 1172 m in 2018. The amount of daily rainfall in the Google Earth Engine (GEE) was calculated using daily CHIRPS data from 2014 to 2018 ^49^. During this time span, the amount of rainfall in the Hashtgerd plain remained constant with no significant change (Fig. 7a). Therefore, precipitation has little effect on groundwater level, which is the average total water level in piezometers. Moreover, there is also a direct relationship between groundwater depletion and land displacement. The land cover map of the plain was used to identify the land use type of areas with the highest rate of subsidence^48^ (Fig. 7b). The spatial distribution of shallow wells and land use types revealed that groundwater reserves in the Hashtgerd plain's center and west are heavily withdrawn for agricultural purposes, causing a decrease in groundwater level and hydrostatic pressure of the aquifer, contributing to subsidence in the region.

**Figure 8:**
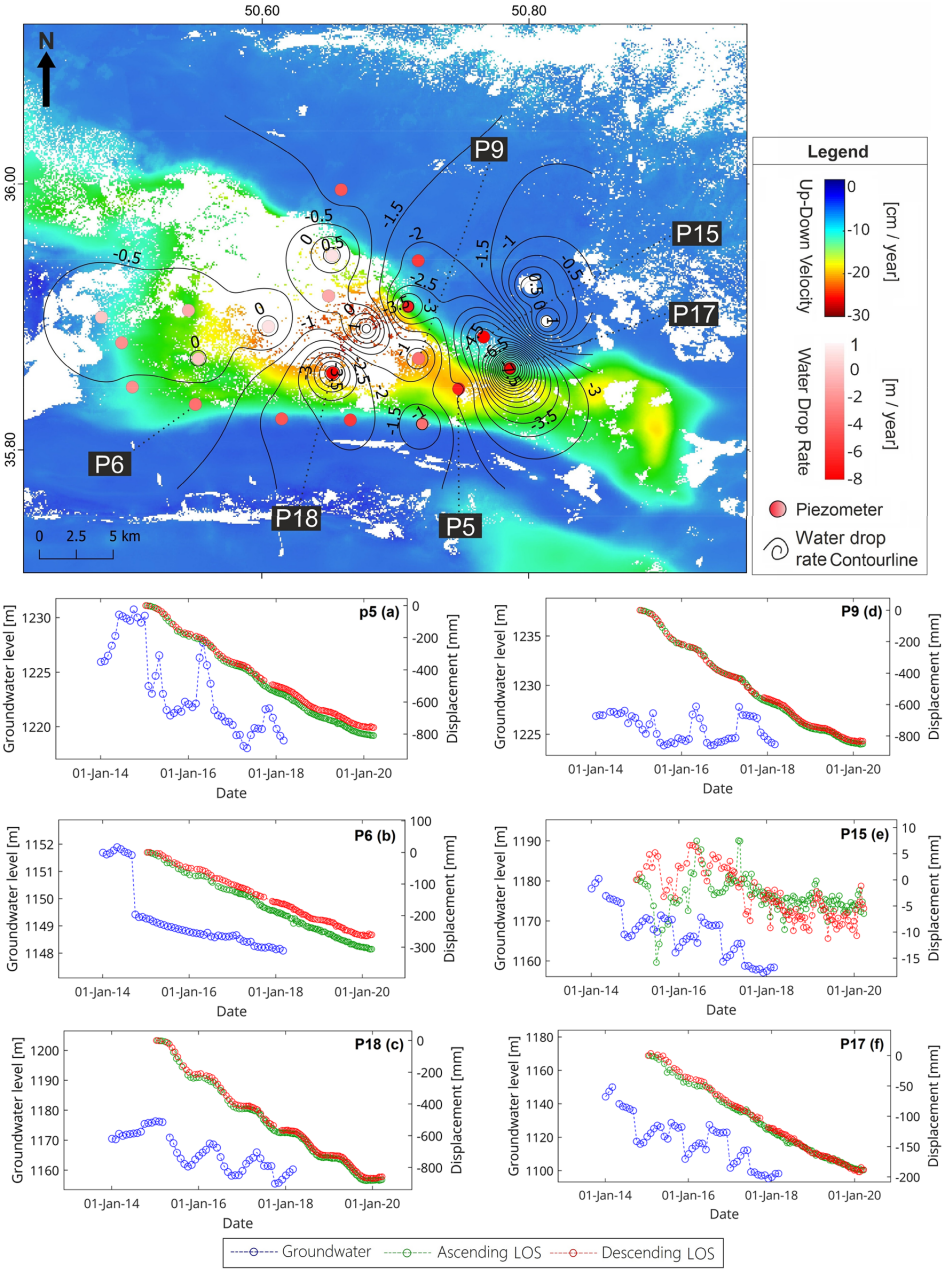
Position and vertical velocity map of piezometer water level variations, comparison of time series of water level of piezometers (blue), and InSAR time series of ascending (green) and descending (red) tracks for piezometers (**a**) P5, (**b**) P6, (**c**) P18, (**d**) P9, (**e**) P17, and (**f**) P15. This figure was created using the QGIS version Desktop 3.18.1 software (https://qgis.org/en/site/).

**Figure 9:**
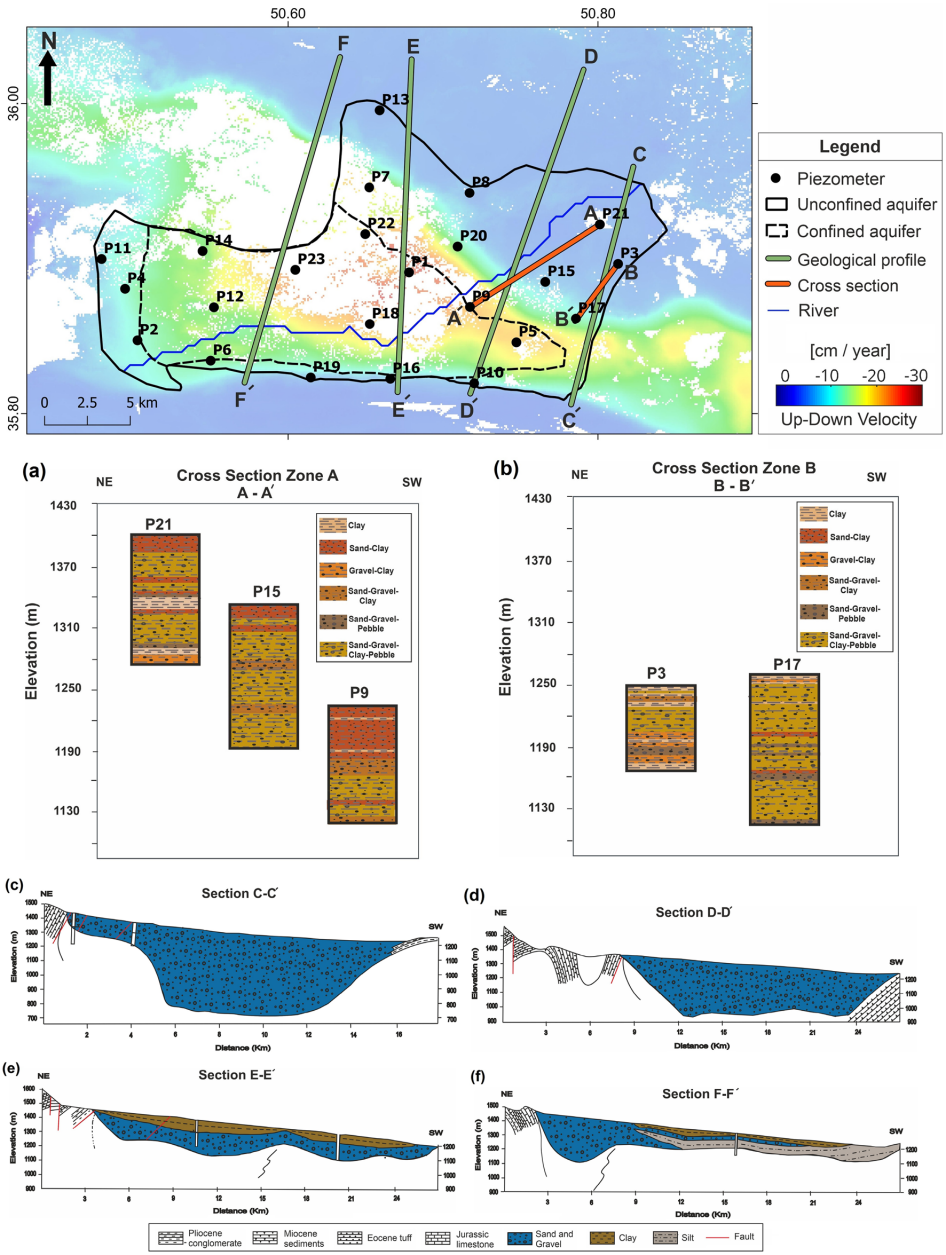
Position of log sections of piezometers and geoelectric sections of confined and unconfined aquifers of Hashtgerd plain for log sections of (**a**) AA’, (**b**) BB', and geoelectric sections of (**c**) CC', (**d**) DD', (**e**) EE’, and (**f**) FF'. This figure was created using the QGIS version Desktop 3.18.1 software (https://qgis.org/en/site/).

This section of the study investigates the link between groundwater fluctuations and subsidence level, as well as the potential impact of geological structures on subsidence. Examination of 23 piezometers with suitable spatial distribution in the region shows that the groundwater level is continuously dropping up to 8 m per year in some wells (Fig. 8). A comparison of InSAR results and the water level of piezometer wells revealed a close relationship between groundwater level drop and subsidence (Fig. 8). In piezometers 5, 6, and 18, the correlation between displacement time series and water loss is 0.98, 0.75, and 0.72, respectively (Fig. 8a–c). For a more detailed investigation of the causes behind subsidence, geological sections obtained from geoelectric data were examined. These wells are located on confined and semi-confined aquifers. Sections CC’ and DD’ show that fine clay and silt sediments become more dominant as we move to the center and west of the plain (Fig. 9a,b). Furthermore, the presence of fine sediment layers in the upper parts of the aquifer has cut the hydrologic connection between the aquifer and the ground surface. The only source of recharge for the semi-confined aquifer is the Kordan River, whose flow has decreased in recent decades, hence, has reduced aquifer recharge. The aquifer's shrinking water level has increased the effective stress and compaction of fine sediments in this area, which is completely consistent with the subsidence pattern.

The water level of piezometers (Fig. 8) shows that the majority of groundwater abstraction has occurred on the unconfined aquifers of alluvial fans in the northern and eastern parts. However, the subsidence rate in these areas is lower in the central areas, and in most cases, is close to zero. Piezometers on alluvial fan unconfined aquifers, such as wells #9 and #15, show no correlation between groundwater level and subsidence. In addition, Piezometer #17 revealed a 0.86 correlation between groundwater depletion and subsidence. The log profile AA' (Fig. 9a), which is located near piezometers #15, #9, and #21, demonstrates that coarse sediments have been deposited alternately in varying proportions. Because coarse sediments with high thickness are most abundant on alluvial fans and grading changes relative to depth are minimal, there is only one layer of unconfined aquifer in this area, and the Kordan River feeds this aquifer in the northeast-southwest direction.

Sand and gravel sediments were more abundant near piezometer #3 on top of the alluvial fan, whereas more gravel, sand, and clay were deposited near piezometer #17 on the eastern margin of the alluvial fan (Fig. 9b). With increasing water abstraction, effective stress and relative compaction have increased, causing little subsidence in this area; thus, the spatial distribution of alluvial fans and thickness of coarse-grained alluvial confirmed the absence of subsidence in the eastern and northern parts of the catchment. The amount of fine-grained clay sediments increases in the sequence of sand and gravel layers as the gradient decreases from alluvial fans to the end of the plain (Fig. 9c–f). The order of sedimentation has formed the confined and unconfined aquifers. Being recharged by the Kordan River and due to the presence of coarse sediments, the unconfined aquifer has little subsidence, but compaction of fine sediments as a result of pumping of confined aquifer wells in the catchment's center and west has created a subsidence bowl area in Hashtgerd plain (Fig. 9).

### Progressive deformation across and along transportation infrastructure

**Figure 10:**
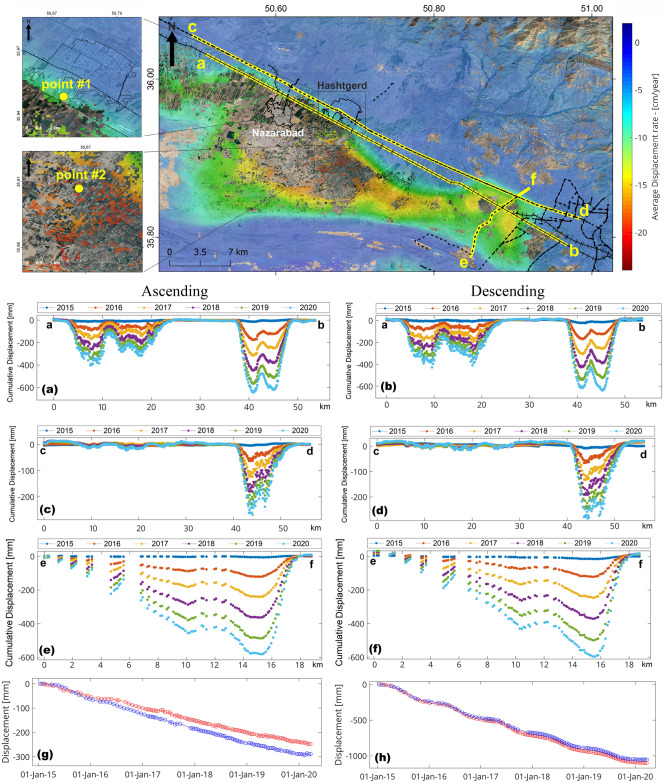
InSAR cumulative displacement for profiles of (**a**,**b**) railway, (**c**,**d**) Karaj-Hashtgerd highway, and (**e**,**f**) profiles crossing the railway and highway, (**g**) a point to the south of Hashtgerd, and (**h**) a point in the area of maximum subsidence in Hashtgerd plain. This figure was created using the QGIS version Desktop 3.18.1 software (https://qgis.org/en/site/).

Figure 10 depicts the position of the profiles drawn along the linear features of the Hashtgerd plain. Profiles are drawn along the railway and the Karaj-Hashtgerd highway can reveal where these linear features are in relation to the cumulative displacement between 2015 and 2020. The ab profile that passes through the railway reveals that the railway line has been affected by subsidence in several locations (Fig. 10a,b). The greatest subsidence has occurred on the railway line between 40 and 45 km, resulting in a cumulative displacement of more than 600 mm in about 6 years. According to the cd profile, the Karaj-Hashtgerd highway has also been affected by subsidence (Fig. 10c,d), with a total displacement of more than 200 mm along this route. The lower amount of subsidence on the highway compared to the railway indicates that as we move away from the subsidence area, the impact of subsidence on linear features decreases, as shown with a line connecting e and f profiles in Fig. 10, which crosses the railway and highway (travel route is from e to f). Furthermore, parts of the Karaj-Qazvin highway have been affected by the subsidence in this area, which could become dangerous as the situation worsens in the coming years. InSAR analysis for an area in the south of Hashtgerd urban zone shows a cumulative displacement of 30 cm between 2015 and 2020. The results show that some areas in the south of the city have been affected by subsidence in recent years, the rate of which is growing (Fig. 10g). Subsidence in this area can be a serious threat to residential areas in nearfuture. Furthermore, the InSAR time series in the center of Hashtgerd plain (Fig. 10h) indicates subsidence of more than one meter in the area over the past 6 years.

Comparing the findings of this study with those of previous investigations in the Hashtgerd plain reveals that the same spatial pattern of subsidence persists. This subsidence pattern has expanded into new areas in the south-east, north-west, and parts of the south of the Hashtgerd city. The results of^27,29^ indicate a maximum subsidence of 14 and 16 cm/year for the years 2003 to 2008 and 2015 to 2020, respectively, indicating an increase in the rate of subsidence.

### Validation of InSAR results

The InSAR results were compared to that of the only available GNSS station. The GNSS station's neighboring pixels with a radius of 300 m were chosen for this purpose. Assuming that horizontal displacements in the GNSS station's range are negligible, the InSAR results in the LOS axis can be converted to vertical displacements using Eq. (2), and the results from the ascending and descending tracks can be compared with the GNSS time series^21,50,51^.

2 $${d}_{LOS}={d}_{U}\mathit{cos}\left({\theta }_{inc}\right)$$

where ${d}_{LOS}$ is the displacement in the LOS axis, ${\theta }_{inc}$ is the incidence angle.

**Figure 11:**
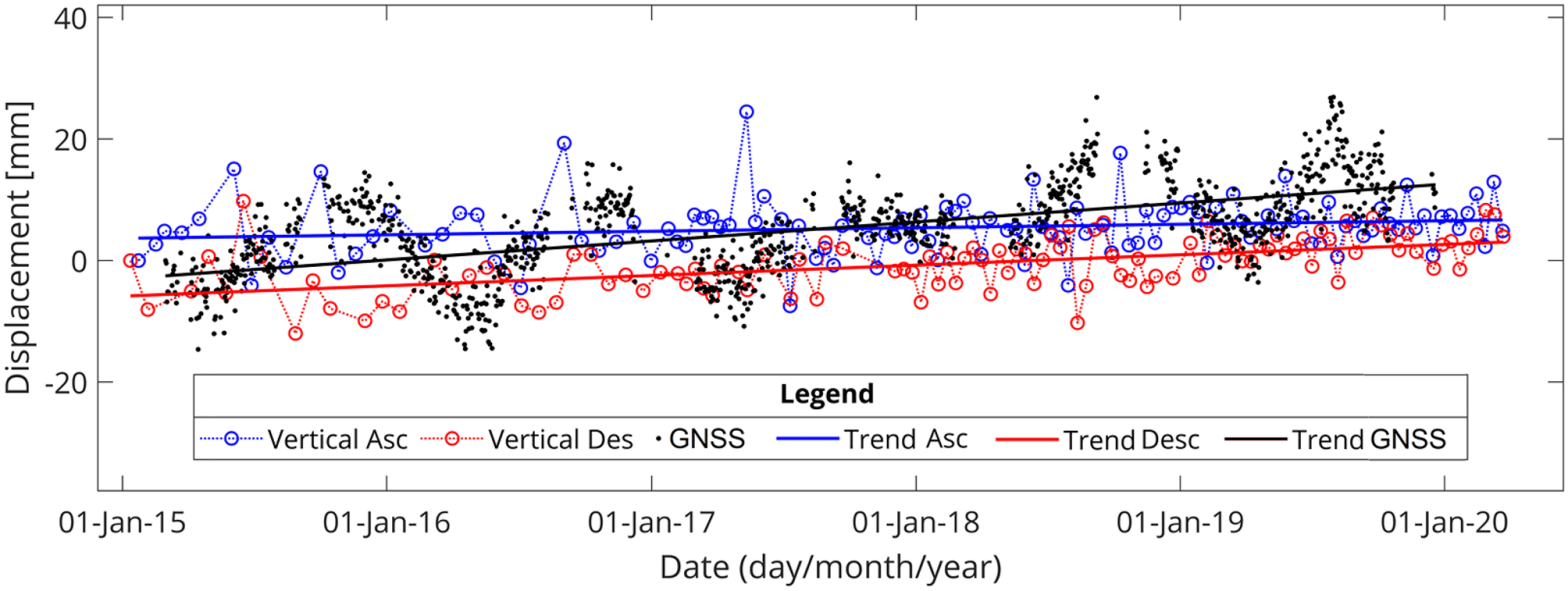
Comparison of vertical displacement of InSAR ascending and descending over time with GNSS station time series.

For both ascending and descending tracks, the displacement value obtained from InSAR time series were compared to that of the GNSS station. The vertical displacement velocity for the GNSS station over a 6-year period was + 2.99 mm/year, and the velocities for ascending and descending tracks were + 0.58 and + 1.76 mm/year, respectively which shows a good agreement (Fig. 11).

**Figure 12:**
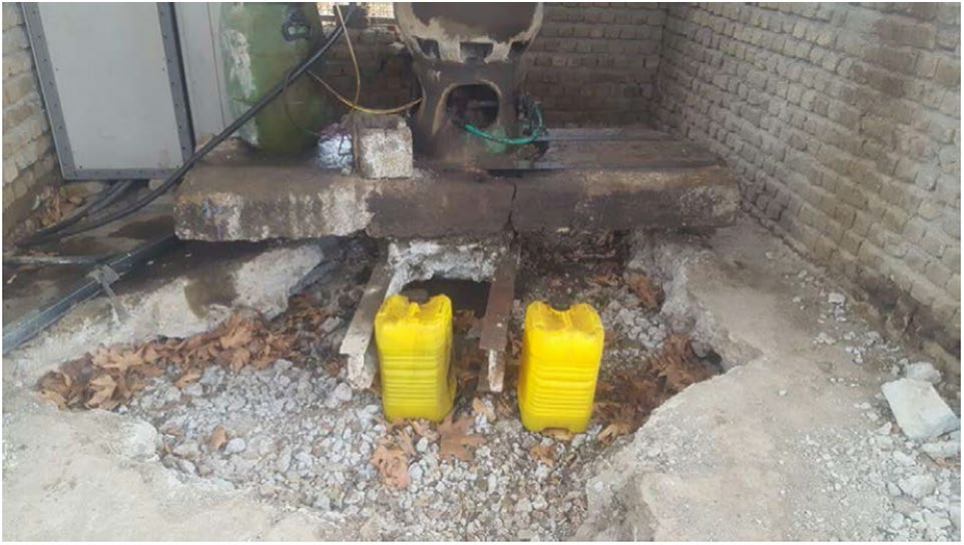
Visible offset of well pipe from the ground surface in Hashtgerd plain which shows a clear subsidence of the ground^52^.

In order to meet the water needs of agricultural activities in this region, deep wells are the most prevalent answer. Another important factor is the permanent operation of these wells which directs groundwater into surrounding lands, leaving no time for the restorage of the water table. When these wells do not produce the required discharge, farmers typically extend their depth, furthering unsustainable groundwater exploitation. Field research conducted by Azizi^52^, illustrates the effects of subsidence on structures. Figure 12 depicts one of these wells which is both the cause and effect of subsidence resulting from excessive groundwater extraction. As demonstrated in Fig. 12, the concrete floor has deteriorated due to changes in the support capacity of the ground surface. The protruding water well in the figure clearly indicates subsidence effects in Hashtgerd plain. It is important to note that the effect of subsidence on these wells is magnified because they are situated in the center of the depression cone beneath them.

## Conclusions

Land displacement in the Hashtgerd catchment was investigated over a ~ 6-year period using Sentinel-1 images and the InSAR technique in both vertical and east–west directions. The maximum subsidence for the ascending and descending tracks is -23 and -22 cm/year, respectively. The central part of the plain has the greatest vertical subsidence, with an annual displacement velocity of about − 30 cm/year. Piezometers and precipitation data were used to explore the connection between groundwater level and subsidence changes in this area. Geoelectric sections and piezometer logs were also used to assess the geological structures of the Hashtgerd catchment. Long-term and uncontrolled groundwater abstraction, type of aquifer, the thickness of fine sediment, the spatial distribution of plain, and alluvial fan were found to be factors contributing to the formation of a subsidence bowl in the Hashtgerd catchment. The number of fine sediments in the sequence of sand and gravel layers increases as the gradient descends from the alluvial fan to the end of the plain. The order of deposition has resulted in two types of unconfined and confined aquifers on the plain and alluvial fan. The unconfined aquifer does not experience much subsidence due to being constantly supplied by the Kordan River and the presence of coarse sediments, but compaction of fine sediments caused by the confined aquifer depletion in the plain's center and west has created a subsidence bowl in Hashtgerd catchment.

The analysis of the profiles along the transportation lines revealed that the subsidence had impacted the sections of the railway and the Karaj-Qazvin highway that pass through this area. Between 2015 and 2020, the cumulative displacement along the railway and highway is − 60 and − 20 cm, respectively. Furthermore, an analysis of the InSAR time series revealed a cumulative displacement of − 30 cm in the southern parts of Hashtgerd. With increased groundwater exploitation for agriculture and horticulture causing a permanent drop in groundwater level in the west and south of Hashtgerd plain, and in the absence of proper recharging of the confined aquifer, the residential areas and transportation lines of the plain will be vulnerable to irreparable damages in the coming years.

## Data Availability

All the hydrological, geological, and GNSS data analyzed during the current study are available as following. The InSAR dataset that used to generate displacement velocity maps are available in the following links: LiCSAR predefined products under the frame ID: 028A_05385_191813, from 2015/01/23 to 2020/04/21 for ascending track [https://gws-access.jasmin.ac.uk/public/nceo_geohazards/LiCSAR_products/28/028A_05385_191813/]; LiCSAR predefined products under the frame ID: 035D_05397_131013, from 2015/01/12 to 2020/04/22 for descending track [https://gws-access.jasmin.ac.uk/public/nceo_geohazards/LiCSAR_products/35/035D_05397_131013/]. The generated datasets along with any other derived results and code scripts that support the findings of this research are available from the corresponding author on reasonable request.
